# Interruption in visual search: a systematic review

**DOI:** 10.3389/fpsyg.2024.1384441

**Published:** 2024-05-14

**Authors:** Alejandro J. Cambronero-Delgadillo, Sarah Jasmin Nachtnebel, Christof Körner, Iain D. Gilchrist, Margit Höfler

**Affiliations:** ^1^Department of Psychology, University of Graz, Graz, Austria; ^2^School of Psychological Science, University of Bristol, Bristol, United Kingdom; ^3^Department of Dementia Research and Nursing Science, University for Continuing Education Krems, Krems an der Donau, Austria

**Keywords:** interruption, systematic review, visual search, visual cognition, interrupting event

## Abstract

Visual search, the process of trying to find a target presented among distractors, is a much-studied cognitive task. Less well-studied is the condition in which the search task is interrupted before the target is found. The consequences of such interruptions in visual search have been investigated across various disciplines, which has resulted in diverse and at times contradictory findings. The aim of this systematic review is to provide a more cohesive understanding of the effects of interruptions in visual search. For this purpose, we identified 28 studies that met our inclusion criteria. To facilitate a more organized and comprehensive analysis, we grouped the studies based on three dimensions: the search environment, the interruption aftermath, and the type of the interrupting event. While interruptions in visual search are variable and manifest differently across studies, our review provides a foundational scheme for a more cohesive understanding of the subject. This categorization serves as a starting point for exploring potential future directions, which we delineate in our conclusions.

## Introduction

Imagine you are driving on an unfamiliar highway, on your way to a dinner party, while searching for your exit from the main road you are on. Your gaze shifts between the road and the overhead signs, trying to match them with the exit number you have memorized. Just when you think you have spotted your exit in the distance, a ring of your phone interrupts your focus. You briefly glance at your phone and discover it is not important. This interruption, although brief, causes you to miss your exit, disrupting your journey and increasing the likelihood of being late for the party.

The scenario above, searching for your exit, exemplifies visual search, the act of locating a target among distractors. Visual search is a common daily behavior; for example, recognizing a street sign in a busy cityscape. Experimentally, this behavior has been explored through various tasks like finding a target letter among a set of other letters (Horowitz and Wolfe, 1998; Höfler et al., 2014), identifying a word on a list (Lawrence, 1971; Radhakrishnan et al., 2022), or locating one specific image among a collection of images (Yang and Zelinsky, 2009; Höfler and Hübel, 2018; Stankov et al., 2021).

Traditionally, interruptions have been conceptualized as temporary cessations of a primary task, typically to divert attention to an alternative task, with the anticipation that the original task will be resumed at a later time, usually once the alternative task is resolved (Boehm-Davis and Remington, 2009). Moreover, since the first pioneering works studying the effect of interruptions (Freeman, 1930), the literature has consistently emphasized the prevailing negative impacts of interruptions on the interrupted task. For our review, we chose to employ a wider definition of interruption: an event that disrupts the search task without necessarily terminating it or initiating a new one.

Scientific research aimed at understanding the impact of interruptions on search tasks has employed a variety of methodologies. These include conducting experiments where participants search for a specific letter and are interrupted by a memory recall task (Beck et al., 2006), interrupting their search with a secondary task under simulated real-world conditions (Kujala, 2013), and naturalistic observation of interruptions in real-world settings (Cades et al., 2010). In the realm of experimental research on interruptions, it is common to let participants resume the interrupted task after interruption. However, in cases of frequent or extended interruptions, returning to the original task might involve substantial cognitive costs and require reengagement of attentional and memory processes (Hirsch et al., 2023). Furthermore, there might be instances where resumption of the original task becomes impossible due to the nature of the interruption. For example, interruptions such as time constraints can make resuming the original task unfeasible due to the irreversible loss of the allocated time for task completion (Höfler et al., 2011; Nachtnebel et al., 2023).

Literature on interruptions often underscores their negative impact on task performance, such as reduced accuracy (Wynn et al., 2018) and longer completion times in the interrupted task (Crews and Russ, 2020). However, it is important to note that interruptions are not necessarily detrimental, and that this prevailing characterization overlooks instances where interruptions may be beneficial. In some instances, well-timed interruptions can enhance productivity by providing necessary mental breaks, thereby improving focus and creativity upon task resumption (Mark et al., 2014). Moreover, interruptions in the form of queries, where additional information is actively sought out, might be necessary for the successful completion of the originally interrupted task (Jin and Dabbish, 2009).

Although the role of interruptions in visual search has sparked some scholarly interest, the subject does not form a cohesive field. Instead, research is spread across various disciplines, each contextualized within its broader domain. For instance, this topic has been explored in the fields of human-machine interaction (Brazzolotto and Michael, 2021), medical imaging (Drew et al., 2018), airport security (Rieger et al., 2021), and basic research in cognitive psychology (Lleras et al., 2005). Despite this fragmented landscape, to the best of our knowledge, no current studies aim to consolidate the scattered insights on the impact of interruptions in visual search. This lack of synthesis underscores the need for a review that collects and consolidates these diverse findings. Therefore, our objective is to present a review that acts as a foundational step in fostering a more cohesive understanding of this subject. By integrating these isolated works, we aim to work toward a unified perspective on how interruptions affect visual search tasks.

Grasping the nuances of interruptions in visual search is vital not only for academic research but also for its practical applications in fields as diverse as defense (Rice and Trafimow, 2012), healthcare (Williams and Drew, 2017) and digital interface design (Kujala and Saariluoma, 2011). In these areas, understanding how interruptions influence visual search can lead to significant improvements in performance and safety. Through our work, we intend to highlight current knowledge gaps, and propose potential avenues for future research. We hope our review will not only spark interest but also inspire further studies. This, in turn, would contribute to the advancement of our understanding of the consequences of interruptions in visual search.

## Methods

We employed a systematic review methodology, conforming to the guidelines of the PRISMA framework (Moher et al., 2009). Our literature search was conducted in October 2023, across the following databases: APA PsycNet, IEEE Xplore, PubMed, Sage Journals, ScienceDirect, Scopus, SpringerLink, and Web of Science. We used the keywords “visual search” AND (“interruption” OR “interruptions”) as queries. We focused exclusively on peer-reviewed articles from scholarly journals and did not apply any publication year filters. The initial results were uploaded to EndNote (The EndNote Team, 2013) for duplicate checking and subsequently imported into Rayyan (Ouzzani et al., 2016), an online tool designed for abstract screening.

After removing duplicates, the initial set of 167 records was reduced to 109. These remaining abstracts were further evaluated for eligibility by AJCD, SJN, and MH based on pre-established inclusion criteria: (i) the study employed an experimental approach; (ii) the paper was authored in English; (iii) adult human subjects participated in the research; and (iv) no clinical samples were part of the study. Additionally, it is important to note that, while some of the studies reviewed included experiments without interruptions, our analysis concentrated exclusively on experiments that deliberately incorporated interruptions into their design. Following the application of these inclusion criteria, resulting discrepancies among the authors were resolved through further discussion.

**Figure 1:**
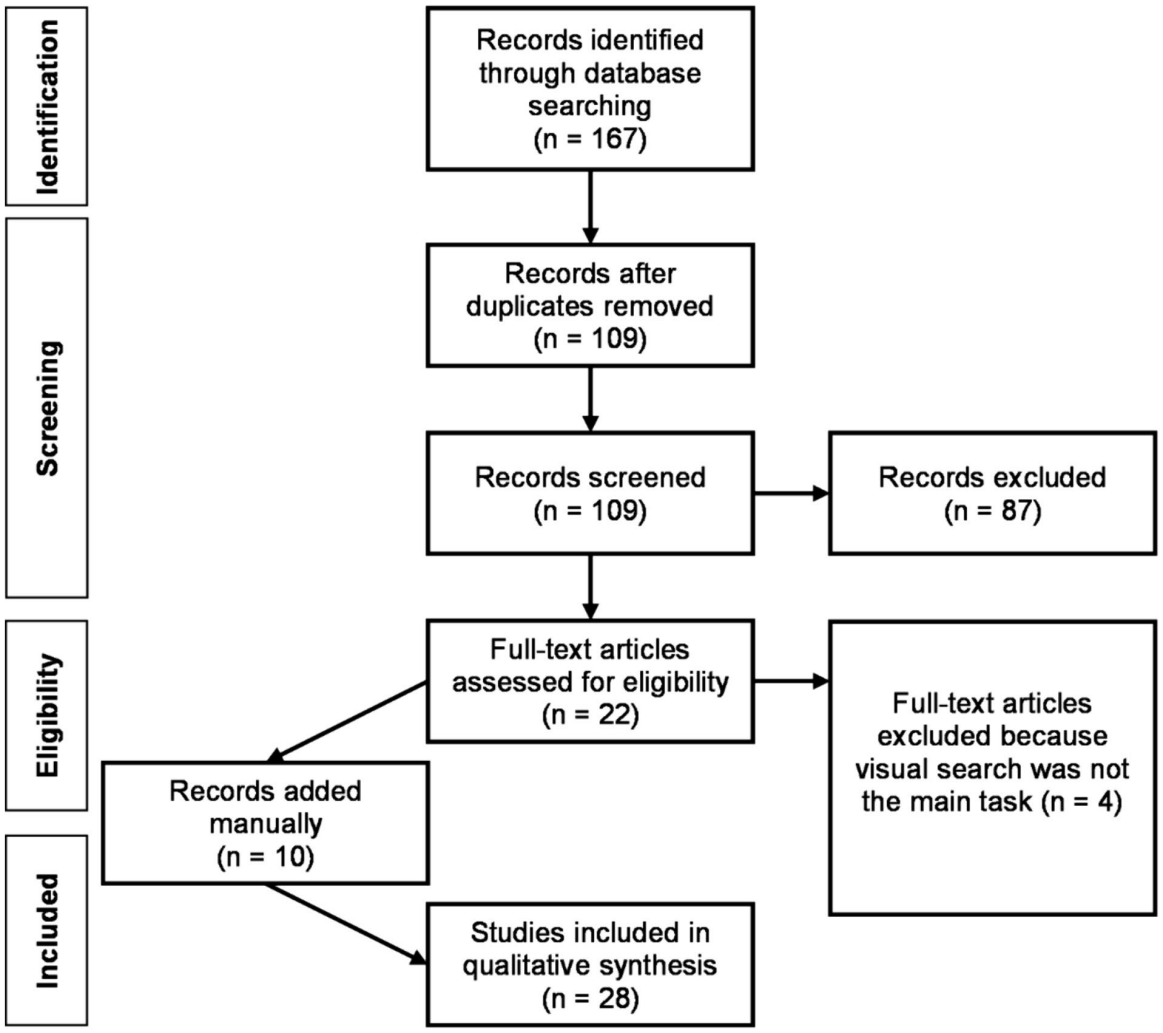
PRISMA flow diagram for the study selection procedure.

Twenty-two articles were initially selected for full-text review. Following the comprehensive full-text review, four of these articles were excluded because visual search did not constitute the main experimental task of the experiments. Additional manual searches, which involved examining the reference lists of these selected articles, led to the identification of ten more articles for potential inclusion. The final corpus for review comprised 28 articles. A PRISMA flow diagram (Moher et al., 2009) depicting the review process is shown in Figure 1.

## Results

We collected and compiled data from each of the chosen studies, including the reference, sample size, country of data collection, main search task, implementation of interruption, interruption onset, interruption frequency main results, all of which are detailed in Table 1. The sample size ranged from 1 to 150 participants. Half (14) of the studies reviewed were conducted in the United States, followed by seven in Canada, two in Austria, two in France, two in Germany and one in the United Kingdom.

**Table 1:** Synopsis of the included studies in alphabetical order.

Code	Reference, sample size (n) and country of data collection	Main search task	Implementation of interruption	Interruption onset	Interruption frequency (%)	Results
A1	Godwin et al. (2013) Experiment 1: 12 Experiment 2: 12 United Kingdom	Participants had to search for a T-shaped target among rotated ‘T’ and ‘L’ distractors and indicate its presence. In Experiment 2, half of the distractor shapes were yellow, differing from the all-blue distractors in Experiment 1.	Interruptions were randomly introduced at pre-determined time intervals, during which the display was filled with a solid color. The interruption lasted 1,200 to 1,400 ms.	100–900 ms after first saccade in search display	80	In interrupted trials, participants exhibited an increased fixation duration, decreased fixation frequency, and diminished fixation-targeting accuracy compared to control trials (Experiment 1). Moreover, participants continued their fixation plans during interruptions, as evidenced by their ongoing saccades and fixations, often revisiting previously observed locations during the interruption phase (Experiment 2).
A2	Jungé et al. (2009) Experiment 1: 26 Experiment 2a: 12 Experiment 2b: 12 Experiment 3: 12 United States	Participants searched for a ‘T’ among ‘L’s and reported its orientation with a key press.	The search display was regularly interrupted by an ‘off’ period, which consisted of a 1,600 ms blank screen. This cycle persisted throughout each trial until a response was made. During the ‘off’ period, distractors could change positions (Experiment 1), orientations (Experiment 2A), or the target’s orientation could change (Experiment 2B). In Experiment 3, half of the items shared the same luminance as the target.	500 ms after search onset	100	Interruptions with distractor changes near the target led to prolonged resumption and slower responses, compared to trials with no change and to those with changes distant from the target (Experiment 1). Additionally, changes in the target’s position during an interruption prolonged search resumption compared to no change (Experiment 2b). Interruptions involving the change of task relevant distractors (i.e., sharing the luminance with the target) disrupted rapid resumption (Experiment 3).
A3	Lleras and Enns (2009) Experiment 1: 16 Experiment 2: 18 Experiment 3: 20 United States	In three experiments, participants searched for a uniquely colored ‘T’ among distractors after looking at a cue. In Experiment 1, the cue was non-predictive of the target’s location. In Experiment 2, the cue always indicated the correct quadrant. In Experiment 3, the cues were placed directly adjacent to the target’s forthcoming location.	Following the cue presentation, epochs consisting of a 100 ms ‘look’ phase followed by a 900 ms ‘wait’ phase were iterated until a response occurred.	100 ms after search onset	100	A nonpredictive spatial cue prolonged the time to identify the target in comparison to trials with no cue (Experiment 1). A spatial cue predicting the target region decreased response times to the target compared to uncued trials. This cue aided rapid resumption, but only after the second look (Experiment 2). A cue in the exact target location greatly sped responses to the target, even more so that in region predicting cues (Experiment 3).
A4	Lleras et al. (2005) Experiment 1: 12 Experiment 2: 12 Experiment 3: 12 Experiment 4: 20 Experiment 5: 18 Experiment 6: 12 United States	In all experiments, participants were tasked with determining the color of a ‘T’ shape among ‘L’ shapes. Experiment 1 involved a display with an equal number of red and blue items. In Experiment 2, participants encountered alternating displays featuring differently colored items. Experiment 3 focused on varying the durations of item displays, while Experiment 4 modified the lengths of blank intervals between displays. In Experiment 5, the search display was presented only once. Experiment 6 involved randomizing item locations with each display appearance.	After a brief presentation of the visual search task (‘on’ period), participants encountered an interruption in the form of a blank screen (‘off’ period), the duration of which varied across experiments. These on–off cycles formed epochs, and the experiments consisted of multiple cycles of epochs.	100 ms after search onset	100	Rapid resumption was only seen after the second epoch onwards (Experiment 1). Rapid resumption was observed in interleaved displays, with participants efficiently resuming interrupted searches rather than starting anew (Experiment 2). Prolonging the on period significantly promoted rapid resumption (Experiment 3). Modifying the duration of the off period did not intervene with rapid resumption (Experiment 4). Target acquisition after a single epoch was proved impossible (Experiment 5). Changing the display configuration between epochs eliminated rapid resumption (Experiment 6).
A5	Lleras et al. (2007) Experiment 1: 15 Experiment 2: 15 Experiment 3: 15 Experiment 4: 15 United States	In four experiments, participants performed a search task to detect a ‘T’ among ‘L’s. In Experiment 1, they indicated the ‘T’s color. Experiment 2 involved randomizing non-target item positions while keeping the target fixed. In Experiments 3 and 4, participants identified the ‘T’s orientation or color, respectively.	In all four experiments, participants encountered a display period lasting 100 milliseconds, followed by an interruption phase (‘wait’) lasting 900 milliseconds. This ‘look-wait’ cycle formed one epoch and repeated until a participant responded or up to 16 times.	100 ms after search onset	100	Rapid resumption was dependent on target-specific preprocessing, that is, when the target was viewed early in the first or second look (Experiment 1). Changing distractor locations between looks reduced rapid resumption slightly (Experiment 2). Target irrelevant features did not disrupt rapid resumption (Experiment 3), whereas target relevant features did (Experiments 4).
A6	Mereu et al. (2014) Experiment 1a: 17 Experiment 1b: 11 Experiment 1c: 17 Experiment 2: 17 Experiment 3: 29 United States	Participants performed a ‘T’ among ‘Ls’ search task involving different target movement patterns: fixed (Experiment 1a), zigzag (Experiment 1b), and random (Experiment 1c). Experiment 2 combined these patterns within the task, while Experiment 3 alternated the target’s orientation between epochs.	Participants were presented with sequences consisting of a 100 ms search display followed by a 900 ms interruption (a blank screen), repeated until a participant responded or up to 12 times.	100 ms after search onset	100	Rapid resumption persisted across interruptions, even when the target’s location changed (Experiments 1a, 1b, 1c). The predictability of the target’s movement did not appear to modulate rapid resumption (Experiment 2). Rapid resumption only emerged when changes in the target’s features were predictable (Experiment 3).
A7	Olds et al. (2000a) Experiment 1: 3 Canada	The experiment consisted of a visual search task in which participants identified a target line with unique orientation amid distractors with varying orientations.	Apart from the control condition, interruptions featured the introduction of a second set of distractors (D2), positioned midway in orientation between the target and the initial distractors (D1).	0–1,000 ms after search onset	100	When the search was interrupted by the early appearance of D2 (> 1,000 ms), participants’ ability to find the target was hindered, resulting in longer response times compared to later appearance of D2.
A8	Olds et al. (2000b) Experiment 1: 7 Experiment 2: 6 Experiment 3: 5 Experiment 4: 4 Experiment 5: 6 Experiment 6: 5 Experiment 7: 6 Canada	Participants engaged in a visual search task involving a display containing a target disk among distractor disks. In each experiment, the task required observers to identify the presence or absence of a target.	Apart from the control condition, a new set of distractors (D2) was introduced after a variable time. Experiment 1 followed a predictable structure with a single timing, while Experiments 2 and 3 intermixed timings. Experiment 4 introduced interruptions by adding identical distractors (D1) to the initial ones on the display. In Experiment 5, trials were intermixed with the addition of identical (D1) or a new set (D2) of distractors. Experiment 6 introduced a color shift in the original distractors during the interruption, and in Experiment 7, when D2 was added, the target was moved to a new position.	0–1,000 ms after search onset	100	Partial pop-out aids difficult search tasks (Experiment 1). Furthermore, this assistance is not reliant on expectations of pop-out process duration (Experiment 2). Partial pop-out aid persists despite uncertainties about the interrupting items (Experiments 3, 4, and 5) and despite the introduction of distractor items that create a non-separable configuration in the pre- and post-interruption display (Experiment 6). Finally, partial pop-out provides spatial location information for visual search. However, when the target moves, this assistance fails (Experiment 7).
A9	Olds et al. (2000c) Experiment 1: 1 Canada	The participant engaged in a visual search task that required discerning a target disk from two sets of colored distractor disks. For each trial, the target, if present, replaced one of the initial distractors.	In every trial, except for the control condition, the search was interrupted with the introduction of a new set of distractors (D2).	0–1,000 ms after search onset	100	In target-present trials, increasing the time before the introduction of D2 (> 200 ms) results in faster response times compared to its earlier introduction.
A10	Olds et al. (2001) Experiment 1: 4 Canada	Participants were asked to locate a target, a yellowish horizontal line, which appeared in 50% of the trials. This target was embedded among distractors consisting of 18 yellowish vertical lines and 18 pinkish horizontal lines.	Except for the control condition, interruptions involved introducing a second set of distractors (D2). In the ‘color singleton’ condition, yellowish vertical distractors were added, and in the ‘orientation singleton’ condition, pinkish horizontal distractors were introduced.	0–1,000 ms after search onset	100	In target-present trials, interruptions led to longer response times when D2 appeared shortly after the search display onset (within 203 ms or less) compared to later appearances. In target-absent trials, response times typically decreased with a longer delay in introducing D2.
A11	Olds and Punambolam (2002) Experiment 1a: 4 Experiment 1b: 3 Experiment 2a: 4 Experiment 2b: 4 Canada	In all experiments, participants were instructed to identify a uniquely colored target disk among distractors disks	Except for the control condition, the search was disrupted by the introduction of an additional set of distractors (D2) after a variable interval. In Experiment 1, D2 was introduced after showing a uniform gray screen, maintaining consistent luminance before and after the interruption. In Experiment 2, D2 was introduced following the display of black disks, resulting in a change in luminance.	0–213 ms after search onset	100	Partial pop-out aids in difficult searches by providing information about the potential target location (Experiment 1a &1b). Altering luminance appears to influence partial pop-out by weakening the information about non-target locations (Experiment 2a & 2b).
A12	Thomas and Lleras (2009) Experiment 1: 18 Experiment 2a: 16 Experiment 3: 16 Experiment 4: 22 United States	In a series of experiments, the persistence of inhibitory tags on items during brief interruptions was investigated. Participants were tasked with locating a T-shaped target among L-shaped distractors, with no target present in half of the trials. Probes in half of the trials tested new or previous item locations. Experiment 2a involved passive observation. Experiment 3 focused attention on half the distractors, while Experiment 4 required participants to identify a target’s orientation and react to a single L-shaped probe.	In each experiment, the search task involved periodic interruptions. The display was shown for 100 ms intervals, followed by 900 ms of a blank display. This pattern continued until the participant’s response. After completing the search task, participants in Experiments 1, 2a, 3, and 4 performed a probe-detection task.	100 ms after search onset	100	Probe detection remained unaffected by the search interruption task, resulting in stable inhibitory tags during short interruptions (Experiment 1 & 3), but not during passive viewing (Experiment 2a) or when the items were unattended during search (Experiment 3).
A13	Van Zoest et al. (2007) Experiment 1: 16 Experiment 2: 12 Experiment 3: 14 Canada	Participants searched for a T-shape among L-shape distractors. In Experiment 2, a contingent gaze paradigm was used: in half of the trials, the target appeared at the participant’s fixation point when the search display reappeared. In Experiment 3, the target was presented at the eye fixation during a specific epoch within the trial.	In all experiments, participants encountered trials with consistent interruptions. Each trial consisted of a 100 ms display followed by a 900 ms blank screen, forming an epoch. This cycle repeated until a response was given. In Experiment 1, participants resumed their search after each epoch. In Experiment 2, they resumed searching with either a standard display or a gaze-contingent display where the target appeared at their eye fixation point.In Experiment 3, the display format depended on the number of elapsed epochs, placing the target at the eye fixation point only after a predefined number of epochs had passed.	100 ms after search onset	100	Interrupting the display caused the participants to need two epochs before rapid resumption was possible. Additionally, responses after a single epoch were slower in contrast to responses following the passage of two or more epochs.
A14	Alonso et al. (2021) Experiment 1: 33 Experiment 2: 31 United States	Participants were asked to find a target among 150 distractor objects. In Experiment 1, the target changed on every trial. In Experiment 2, the target remained consistent across trials. Participants indicated their choice by clicking the target or a designated absent box.	In both experiments, shortly after the display appeared, a red screen with random characters covered the display. Participants were required to type at least 80% of these characters correctly.	500 or 700 ms after search onset	10	In both experiments, interruptions led to longer response times in the search tasks compared to uninterrupted trials. Additionally, these interruptions also reduced search accuracy in interrupted trials compared to uninterrupted trials (Experiment 1).
A15	Labonté and Vachon (2021) Experiment 1: 110 Canada	In a Multiple Object Tracking (MOT) task, participants tracked red target dots among moving dots of various colors for 15–25 s. Afterward, all dots stopped and turned black, and participants had to identify the dots they originally selected as targets.	The interruption consisted of a mathematical verification task consisting of one, three, or six arithmetic equations. During this task, the dots in the MOT task continued to move in the background.	5–30 s after search onset	75	Interruptions reduced the accuracy of target identification compared to uninterrupted conditions. This decline was exacerbated with longer interruptions. Moreover, the duration of the interruption had an effect on resumption lag, with longer interruptions leading to increased lag.
A16	Ratwani and Trafton (2008) Experiment 1: 13 Experiment 1: 36 United States	In both experiments, participants searched through a column of numbers to identify and type odd numbers into a separate ‘copy box,’ starting from the top of the column and moving downward.	In Experiment 1, trials were interrupted by an arithmetic task. In Experiment 2, 25% of trials were interrupted by an arithmetic task, and 25% were interrupted by a spatial mental rotation task.	At the beginning of a trial	50	In both experiments, interruptions led to a longer resumption lag compared to the uninterrupted condition. Additionally, spatial interruptions were more disruptive than nonspatial interruptions (Experiment 2), as evidenced by a greater increase in resumption lag for the former compared to the latter.
A17	Beck et al. (2006) Experiment 1: 20 Experiment 2a: 11 Experiment 2b: 24 Experiment 2c: 25 United States	Participants were required to find a target letter among distractor letters using an oculomotor contingent paradigm, where at most three items from the search set were visible simultaneously. In Experiments 2a, 2b, and 2c, all items were initially concealed by placeholders and were only revealed upon detection of an imminent fixation.	Interruptions consisted of the emergence of a red box at a specific location. This box contained two items: one previously examined (either at this specific location or elsewhere) and a foil. Participants had to choose the item they had fixated on previously (or both the item and its location) from these two options (2AFC).	After 6 fixations in search display	30	Items examined just before the interruption exhibited the highest recall performance. Recall performance declined for items observed earlier. Moreover, participants were generally better at remembering the identities of the items than their exact locations.
A18	Höfler et al. (2011) Experiment 3: 12 Austria	Participants conducted two consecutive searches within the same 15-letter display for different target letters. Probes were presented after the first saccade in the second search to test for the presence of inhibition of return.	The first of the two consecutive searches was interrupted on half of the trials by announcing a new target letter through the loudspeakers. The participants were required to stop searching for the first target and continue searching for the second target.	After fixating 5–9 items	50	Inhibition of return (measured by saccadic responses to a probe) was observed across the two searches if the first search was interrupted while it was not present when the first search was completed.
N1	Brazzolotto and Michael (2020) Experiment 1: 46 Experiment 2: 42 France	Participants engaged in a simulated email sorting task, where they were required to identify target emails among distractors within an inbox. Once all target emails were located, they moved to the next inbox by clicking a ‘trash’ icon.	Participants were interrupted by a working memory task of varying difficulty (memorization and recall of series of numbers). This was followed by a ‘time-before-resumption’ (TBR) period, during which a white screen was displayed.	After selecting 2 or 8 targets	85	Interruptions led to longer resumption lags in trials with interruptions than in those without. Moreover, difficult interruptions resulted in decreased accuracy in the search task and extended the resumption lag compared to easy interruptions. Finally, the introduction of an extended TBR mitigated this resumption lag, but only after a difficult interruption.
N2	Brazzolotto and Michael (2021) Experiment 1: 46 France	Participants had to search for specific emails on a simulated inbox interface. After identifying all targets emails, they moved on to a new inbox by clicking on a “thrash” icon.	The interruption involved the display of arithmetic problems, each presented for three seconds. Simultaneously, a background image with emotional content was gradually revealed based on each participant’s response.	After selecting 1–9 targets	100	Interruptions led to increased task response latency, extending the time needed to resume and respond to the search task post-interruption. Images rated as highly pleasant or highly unpleasant caused the most significant delays, compared to those rated as neutral.
N3	Drew et al. (2018) Experiment 1: 18 Experiment 2: 16 United States	Radiologists were tasked with diagnosing medical images. That could contain (Experiment 1) or did not contain (Experiment 2) critical findings.	In Experiment 1, participants were interrupted by a phone call during the scanning process. This required the radiologists to consult a separate scan and provide a diagnosis. In Experiment 2, participants were interrupted with a demographic survey.	Experiment 1: 3 min after search onset Experiment 2: randomly during search	Experiment 1: 50 Experiment 2: 25	Interruptions resulted in longer search times and reduced accuracy, but only in the first interrupted case (Experiment 1). Interrupted cases were associated with a less thorough examination of critical areas for diagnosis compared to uninterrupted cases. Additionally trial duration remained consistent when the task was unrelated, indicating that interruption-related time costs are dependent on the nature of the task (Experiment 2).
N4	Radović et al. (2022) Experiment 1: 150 Germany	Participants had to find a target letter amid distractor letters in a simulated x-ray image. They were randomly placed into one of three conditions, each differing in interruption frequency: low (25%), medium (50%), or high (75%).	During an interruption, the visual search display was replaced by three numbers, presented sequentially. Participants had to determine whether each number was odd or even.	Randomly based on individual performance	25, 50 or 75	Interrupted trials resulted in slower response times to the target compared to uninterrupted trials. Additionally, a high frequency of interruptions led to faster response times compared to a low frequency of interruptions. Finally, responses in interrupted trials were less accurate (but only in target-absent trials).
N5	Williams and Drew (2017) Experiment 1: 26 Experiment 2: 27 United States	In both experiments, participants conducted visual searches on chest CT scans to locate and mark lung nodules. They used a computer mouse to highlight the identified nodules and ended the search task by clicking a designated box on the screen.	The interruptions consisted of math equations with different levels of difficulty.	30–60 s after search onset	Experiment 1: 50 Experiment 2: 33	Interruptions resulted in longer search times compared to control trials, although the difficulty of the interruption did not significantly affect this increase.
N6	Wynn et al. (2018) Experiment 1: 23 United States	In the main search task, participants assessed chest radiographs categorized into three difficulty levels: normal, subtle, and unsubtle. Their objective was to detect the presence of pneumothorax.	The interruption consisted of a new search task requiring participants to identify the orientation of a ‘T’ among multicolored ‘L’s. This interruption lasted for 30 s.	8 s or 10s after search onset	30	Interruptions increased search times for the main visual search task. Moreover, interruptions impacted accuracy differently depending on the case type: reducing it in subtle cases, improving it in normal cases, and having no effect on unsubtle cases.
N7	Shen and Jiang (2006) Experiment 1: 9 Experiment 2: 20 Experiment 3: 20 Experiment 4a: 15 Experiment 4b: 20 Experiment 5a: 10 Experiment 5b: 10* Experiment 6: 20 United States	In a change detection task, participants had to identify a changing object in alternating displays of two slightly different images, which could either be polygons overlaid on a natural scene or on a gray background. These displays alternated with a solid gray display. Each display lasted 300–400 ms depending on the experiment. The participants’ objective was to detect the changing object in each display.	An unfilled delay of varying time (up to 6 s) with no additional task was presented after predefined cycles of stimulus presentation. After the delay, the re-appearance was either a change in the configuration or shape of the polygons and/or background or no change at all, and participants continued searching for the target (Experiments 1–3). Besides an unfilled and/or no delay, passive viewing and/or active searching was required during the delay. The delay lasted between a couple of seconds up to 3 min across experiments and the display was either partly, fully, or not repeated (Experiments 4–6).	300–400 ms after search onset	100	Search efficiency remained high when polygon layout remained stable, highlighting the importance of spatial item arrangement in memory (Experiments 1–3). However, introducing an extra task during the delay period affected memory and subsequently impacted search performance (Experiments 4–6).
N8	Rieger et al. (2021) Experiment 1: 48 Germany	Participants used a mouse-over procedure to examine X-ray images of luggage for potential threats by moving a rectangle to reveal parts of the image. They performed this task either with assistance from an automated system or without any aid.	Each trial was subject to a potential interruption due to low or high time pressure. If participants did not make a decision before time expired, the response was automatically registered as ‘target absent’.	Countdown starting at search onset	100	High time pressure decreased response accuracy and led to quicker, less thorough searches, resulting in faster response times compared to the low time pressure condition.
N9	Nachtnebel et al. (2023) Time Pressure Condition: 14 Austria	Participants were guided to eight distinct locations within a university campus to engage in a visual search task in the real world. Each location required participants to identify 10 target objects (half of them present) by means of a button press on a tablet.	In one of four experimental conditions (time pressure condition), participants were informed they might not be able to complete their search in some trials. These trials were interrupted by a tone, which was then followed by a mathematical equation that participants needed to classify as correct or incorrect.	9–13 s after search onset	45	Time pressure did not seem to affect search accuracy compared to non-interrupted experimental conditions. This suggests that participants may have adapted their behavior in response to the time constraint.
N10	Rice and Trafimow (2012) Experiment 1: 16 United States	The participants’ task was to identify targets in aerial photos using a diagnostic aid that provided guidance on the presence of targets.	Half of the trials were interrupted after 2 s (speeded), while the other half were interrupted after 8 s (unspeeded) by a screen prompting participants to respond to the target’s presence.	Countdown starting at search onset	100	In the speeded condition, participants showed higher response accuracy and more consistent responses compared to the unspeeded condition.

The impact of interruption on the visual search task was evaluated using metrics commonly employed in visual search paradigms. These included search accuracy and response time, along with measures of oculomotor behavior, such as the duration and frequency of fixations. Additionally, specific metrics designed for experimental paradigms involving interruptions, like resumption lag (i.e., the time taken to resume the search task after the interruption), were utilized.

**Figure 2:**
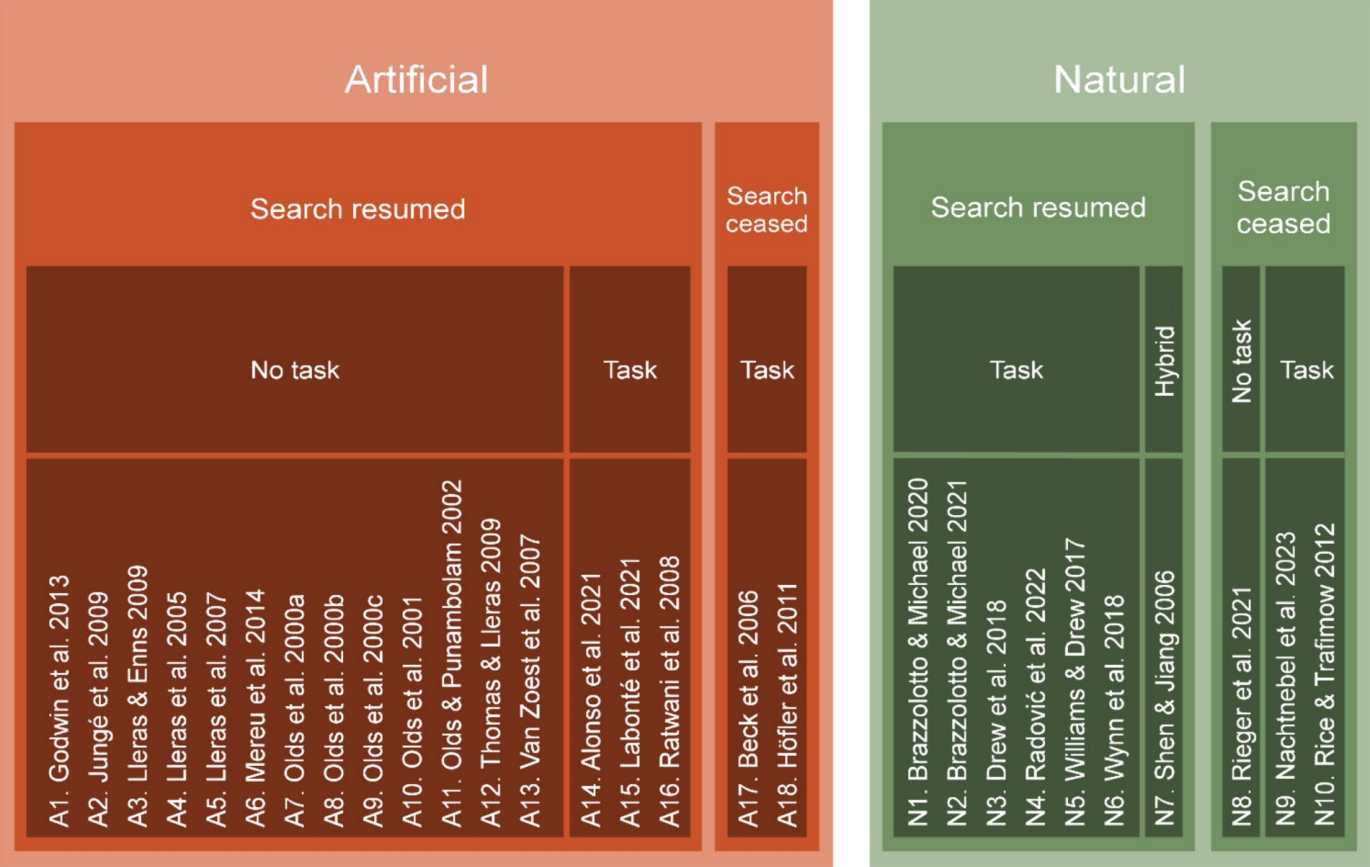
Categorization scheme by search environment (artificial vs. natural), interruption aftermath (search resumed vs. search ceased), and interrupting event (task vs. no task).

To categorize the reviewed studies, we employed a hierarchical scheme based on three key aspects (Figure 2). At the first level, studies were categorized based on the search environment, distinguishing between “artificial” environments, common in laboratory visual search studies (18/28 papers), and “natural” environments, representing those utilizing more ecologically valid search settings (10/28 papers). The second level addresses the interruption aftermath, specifically, whether the original search was resumed or ceased after the interruption. Most studies reviewed (22/28) implemented interruptions that disrupted the search task only momentarily, allowing participants to resume the search afterwards. The remaining six studies investigated scenarios where interruptions led to a complete cessation of the search, rendering it impossible for participants to resume their search post-interruption. Finally, the third level classifies studies based on whether the interrupting event necessitated participant action: task-required events (13), such the appearance and subsequent solving of an arithmetic task (Nachtnebel et al., 2023), non-task events (14), such as the brief disappearance of the search display (Thomas and Lleras, 2009), and hybrid events (1), which involve a combination of action-required and non-action-required events (Shen and Jiang, 2006).

**Figure 3:**
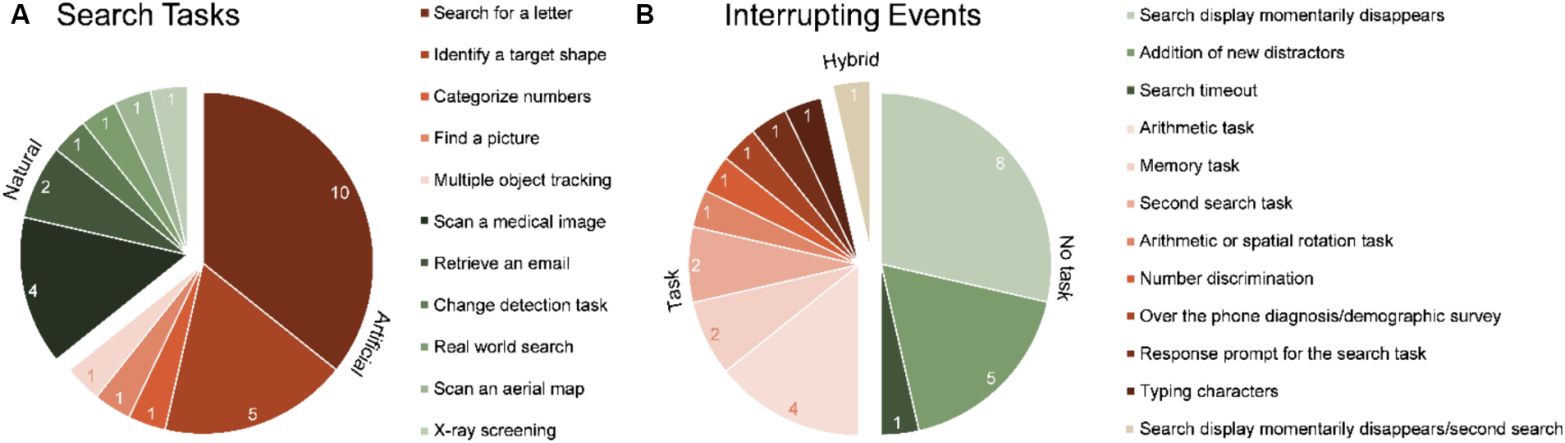
Comparative distribution of search tasks **(A)** and interrupting events **(B)** present in the reviewed studies.

Figure 3A illustrates the distribution of search tasks utilized in the reviewed studies. A majority of research in artificial environments (10/18) was conducted with the task of identifying a specific letter among distractors (Lleras et al., 2005, 2007; Beck et al., 2006; Van Zoest et al., 2007; Jungé et al., 2009; Lleras and Enns, 2009; Thomas and Lleras, 2009; Höfler et al., 2011; Godwin et al., 2013; Mereu et al., 2014). Furthermore, five of the studies asked participants to identify a target shape (Olds et al., 2000a,b,c, 2001; Olds and Punambolam, 2002) while one study each explored finding a picture (Alonso et al., 2021), categorizing numbers (Ratwani and Trafton, 2008) and tracking multiple objects (Labonté and Vachon, 2021). In contrast to the more homogenous distribution of search tasks in studies conducted in artificial environments, natural environment search tasks exhibited greater heterogeneity. They encompassed four medical image scanning tasks (Williams and Drew, 2017; Drew et al., 2018; Wynn et al., 2018; Radović et al., 2022), one change detection task in natural scenes (Shen and Jiang, 2006, all experiments except 5b), one search in the real world (Nachtnebel et al., 2023),one scanning an aerial map (Rice and Trafimow, 2012), and one X-ray screening task (Rieger et al., 2021).

Regarding the interruption aftermath, in most of the studies conducted in artificial environments (13/18), participants typically were allowed to continue their search after an interruption without needing to complete additional tasks (Olds et al., 2000a,b,c, 2001; Olds and Punambolam, 2002; Lleras et al., 2005, 2007; Van Zoest et al., 2007; Jungé et al., 2009; Lleras and Enns, 2009; Thomas and Lleras, 2009; Godwin et al., 2013; Mereu et al., 2014). However, in three studies, participants were required to perform another task following the interruption before they could resume their search (Ratwani and Trafton, 2008; Alonso et al., 2021; Labonté and Vachon, 2021). Additionally, in two instances where the interruption marked the end of the search, a further task followed the interruption (Beck et al., 2006; Höfler et al., 2011).

Most studies carried out in natural environments (6/10) allowed for search resumption, however this was always dependent on the completion of a task during the interruption (Williams and Drew, 2017; Drew et al., 2018; Wynn et al., 2018; Brazzolotto and Michael, 2020, 2021; Radović et al., 2022). In natural scenarios where the interruption ended the search, participants were required to complete a task in two studies (Rice and Trafimow, 2012; Nachtnebel et al., 2023) while in one study, they were not required to perform any task post-interruption (Rieger et al., 2021). Finally, the study by Shen and Jiang (2006) exhibited a hybrid pattern: in experiments 1–3, completing a task was not required to resume the search, while in experiments 4–6, it was necessary.

When we assessed whether the interruption event was considered a task or not, we observed that 14 studies did not involve a task as the interruption (Olds et al., 2000a, 2000b, 2000c, 2001; Olds and Punambolam, 2002; Lleras et al., 2005, 2007; Van Zoest et al., 2007; Jungé et al., 2009; Lleras and Enns, 2009; Thomas and Lleras, 2009; Godwin et al., 2013; Mereu et al., 2014; Rieger et al., 2021); and all but one of these studies (Rieger et al., 2021) were conducted in artificial environments. In contrast, in the 13 studies where the interruption was a task, five were conducted in artificial environments (Beck et al., 2006; Ratwani and Trafton, 2008; Höfler et al., 2011; Alonso et al., 2021; Labonté and Vachon, 2021) and eight in natural ones (Rice and Trafimow, 2012; Williams and Drew, 2017; Drew et al., 2018; Wynn et al., 2018; Brazzolotto and Michael, 2020, 2021; Radović et al., 2022; Nachtnebel et al., 2023). Within these 13 studies, two involved interrupting event tasks that were directly related to the interrupted search, requiring participants to use information obtained during the incomplete search to complete their tasks (Beck et al., 2006; Rice and Trafimow, 2012) whereas the tasks in the remaining nine studies were search-unrelated.

Overall, artificial environments exhibited a more limited variety of interrupting events compared to natural settings. The distribution of these events is depicted in Figure 3B. In artificial environments, seven types of events were observed, five of which were unique to these settings. Conversely, natural settings featured eight different types of events, with six exclusive to them. Tasks specific to artificial environments included the momentarily disappearance of the display (Lleras et al., 2005, 2007; Van Zoest et al., 2007; Jungé et al., 2009; Lleras and Enns, 2009; Thomas and Lleras, 2009; Godwin et al., 2013; Mereu et al., 2014), the addition of new distractors to the display (Olds et al., 2000a,b,c, 2001; Olds and Punambolam, 2002), a new search in the same display (Höfler et al., 2011), typing characters (Alonso et al., 2021), or a hybrid task requiring either and arithmetic or a spatial rotation task (Ratwani and Trafton, 2008). Tasks unique to natural environments included number discrimination (Radović et al., 2022), a prompt to respond to the interrupted search (Rice and Trafimow, 2012), a search for a letter (Wynn et al., 2018), the timeout and accompanying end of the search (Rieger et al., 2021), a hybrid task requiring either an over the phone diagnosis or the completion of a demographic survey (Drew et al., 2018) or an event that was either the momentary disappearance of the search display or passive viewing of a natural scene (Shen and Jiang, 2006). Finally, two interrupting events where common to both environments: arithmetic tasks (Williams and Drew, 2017; Brazzolotto and Michael, 2021; Labonté and Vachon, 2021; Nachtnebel et al., 2023) and a memory tasks (Beck et al., 2006; Brazzolotto and Michael, 2020).

In considering the effects of interruption on visual search tasks, we observed that most studies indicate a disruptive impact. However, there was extensive variation in the nature and extent of this disruption. To provide a thorough analysis, we focused on the distinct aspects of the search process influenced by these disruptions. The two most prominent findings are that interruptions impacted search accuracy and search times, as evidenced by reduced accuracy and extended response times in interrupted trials compared to uninterrupted ones (Williams and Drew, 2017; Alonso et al., 2021; Rieger et al., 2021). The timing of the interruption emerged as a key factor influencing the extent of search disruption. Notably, the first interruption led to longer response times and reduced accuracy (Drew et al., 2018) as compared to subsequent interruptions (Van Zoest et al., 2007). In this same vein, the early onset of interruptions resulted in longer response times (Olds et al., 2000c) compared to later onsets (Olds et al., 2000a, 2000b, 2001).

The impact of interruptions on search resumption was influenced by the nature of the interrupting event. Longer (Labonté and Vachon, 2021) and more difficult (Brazzolotto and Michael, 2020) interruptions significantly delayed the resumption time compared to shorter or simpler ones. Similarly, interruptions involving emotionally charged stimuli extended resumption time more than neutral interruptions (Brazzolotto and Michael, 2021). Additionally, spatial interruptions caused greater resumption lag than non-spatial ones (Ratwani and Trafton, 2008). Furthermore, the ability to quickly resume the search after an interruption, known as rapid resumption (Lleras et al., 2005), was adversely affected when interruptions involved changes to task-relevant distractors that shared features with the target (Jungé et al., 2009). However, changes to task-irrelevant distractors (Lleras et al., 2007) or the target’s location did not hinder rapid resumption (Mereu et al., 2014).

Memory, which is crucial in visual search (Hollingworth, 2006), appears to be disrupted by interruptions. When testing for recall performance, items observed shortly before an interruption were remembered best, with recall declining for items observed earlier (Beck et al., 2006). Memory for probes seemed to be robust to short interruptions but not too long ones (Thomas and Lleras, 2009). Interruptions involving moving to a new task seemed to disrupt spatial item arrangement in memory, consequently resulting in decreased search performance (Shen and Jiang, 2006). Further supporting this, participants needed more time to resume their search tasks after an interruption involving a spatial task interruption, compared to non-spatial task interruptions (Ratwani and Trafton, 2008).

Interruptions during the search task also impacted participants’ oculomotor behavior. These interruptions significantly influenced measures such as fixation duration (increased), fixation frequency (decreased) and diminished fixation-targeting accuracy (Godwin et al., 2013). Interestingly, when conducting two consecutive searches, inhibition of return, the phenomenon where individuals exhibit delayed reactions to a stimulus appearing at a location they have recently examined (Klein, 2000), was observed when the first search was interrupted, however, this inhibition was not present in scenarios where the first search was completed without interruption (Höfler et al., 2011).

Even though interruptions were generally detrimental to the ongoing visual search, some studies have provided conflicting or outright contradictory evidence, particularly in natural environments. For example, Nachtnebel et al. (2023) observed no significant difference in accuracy between interrupted and uninterrupted search conditions in a real-world setting. Additionally, some interruptions in the form of time pressure (i.e., the participant has a limited time to respond) have shown to improve response accuracy in computer-assisted searches (Rice and Trafimow, 2012) and to speed up responses without changes in accuracy (Radović et al., 2022). Furthermore, the impact of interruptions on search accuracy may vary depending on the task difficulty. For instance, Wynn et al. (2018) found that difficult searches (i.e., targets with inconspicuous features) were negatively affected by interruptions involving a new task, while easier searches (i.e., targets that are less difficult to spot) generally showed improved accuracy. In artificial environments, interruptions providing spatial information about the target’s location have been shown to decrease response times (Olds et al., 2000a; Olds and Punambolam, 2002; Lleras and Enns, 2009).

## Discussion

The aim of this review was to provide a comprehensive synopsis of existing research on interruptions in visual search. It underscored the importance of identifying and bridging knowledge gaps within this field, aiming to create a more unified and thorough understanding of the phenomenon. The primary observation is the lack of uniformity in defining and executing interruptions, evident in the methodological diversity of studies. While methodological pluralism diversifies the field with a wide array of insights and perspectives, it complicates the development of a unified understanding of interruption effects in visual search, thereby hindering their integration into existing cognitive models.

Our review encompassed a range of studies from different disciplines. While not every study included was primarily focused on the effect of interruption in visual search, each integrated the aspect of interruption into its methodology, which became the central focus of our analysis. We adopted this approach with the intention of capturing the diverse ways in which interruptions manifest in visual search tasks. In doing so, we found that the diversity and complexity of these studies presents a substantial challenge for conducting a structured analysis. As a starting point, we therefore defined interruption as an event that disrupts the search task without necessarily terminating it or initiating a new search. Following this definition, we constructed a categorization scheme focused on three critical dimensions: the search environment, the aftermath of the interruption, and if the interrupting event prompted a task.

In our review, we observed that studies conducted in artificial environments often employed well-established search tasks prevalent in the visual search field, such as finding a letter among distractors or identifying a target shape. The use of methodologically rigorous and tested paradigms facilitates experimental control and enables comparisons across different studies. However, this approach may compromise ecological validity, and poses a challenge in the translation of findings to practical applications (Diaz et al., 2003). In contrast, research conducted in natural environments tends to reflect real-world search scenarios more accurately. However, these naturalistic studies often employ diverse methodological approaches, even within the same subdiscipline (Ratwani et al., 2016), leading to results that are challenging to compare across studies. Consequently, the findings are less straightforward, necessitating careful contextualization and cautious interpretation.

Regardless of the search environment, in the majority of reviewed studies participants were allowed to resume search after the interruption, which is in line with the classical definition of interruption, which conceptualizes them as the temporary cessation of a primary task (Boehm-Davis and Remington, 2009). In real-life, we usually have the opportunity to return to our initial tasks after being interrupted. As such, this implementation of interruptions reflects real-world occurrences, where the process involves a pause followed by a continuation. For instance, imagine searching for a book in a library and being interrupted by a phone call; after the call, you would typically resume your search for the book. For the studies in which the interruption marked the end of the search, the interrupting event was always triggered by the expiration of allotted time (Rice and Trafimow, 2012; Rieger et al., 2021; Nachtnebel et al., 2023) or after a predetermined number of saccades (Beck et al., 2006; Höfler et al., 2011). This mirrors real-life scenarios where searches are often time-constrained, such as when we are shopping in a supermarket before it closes. Interestingly, none of the studies incorporated endogenous (i.e., participant-initiated) interruptions, which are common in daily life. For example, if we are searching for a rain jacket before an appointment and cannot find it quickly enough, we might switch our search to looking for an umbrella instead. This type of internally driven interruptions represents an area for further research.

Concerning interrupting events, clear differences were observed between artificial and natural settings. In artificial environments, interrupting events were typically predictable and consistently timed, with the aim to test clearly defined effects such as rapid resumption (Lleras et al., 2005). These interruptions often did not involve additional tasks; they were merely brief disappearances of the search display, followed by the reappearance of the same or a slightly altered display. Conversely, in natural environments, most interruptions occurred unpredictably and involved an additional task. Furthermore, the tasks prompted by these interruptions were organically connected to the preceding interrupted search, meaning they could realistically occur in such contexts—for example, a physician receiving a phone call from a patient while scanning a medical image (Drew et al., 2018). These interruptions in natural settings were designed to explore how interruptions could operate in real-world scenarios, providing valuable insights into their practical impacts.

As previously mentioned, research on the effects of interruptions has consistently emphasized their predominantly adverse consequences. Indeed, interruptions were accompanied by negative effects on the search process in all the studies included in our review, predominantly manifesting as reduced accuracy and extended response times. Interestingly, we also found that search planning was affected by search interruption. For instance, Godwin et al. (2013) observed that participants briefly continued their fixation plan during the interruption period, as evidenced by their saccades and fixations often revisiting locations observed prior to the interruption. Moreover, Höfler et al. (2011) reported that inhibition of return, i.e., a phenomenon where attention is less likely to return to a previously attended location (Klein, 2000), persists across two consecutive searches when the initial search was interrupted but it extinguishes when the initial search was completed. Nevertheless, under specific conditions, interruptions might carry a beneficial impact on visual search. For instance, Ratwani et al. (2006) demonstrated that interrupting a simple search resulted in shorter fixation durations and fewer task-critical errors compared to uninterrupted searches. Similarly, Rice and Trafimow (2012) observed that participants achieved greater accuracy in their responses when they were under time pressure compared to a control condition without such constraints. Thus, studying interruptions in visual search not only helps understand their immediate effects but also provides insights into broader aspects of search dynamics, potentially guiding the development of methods to either mitigate or capitalize on these effects.

While there are clear indications of potential benefits, the underlying mechanisms and specific contexts from which interruptions are beneficial also require further investigation. We suggest that future work in investigating interruptions in visual search tasks could draw from the methodologies and theories from studies identifying advantageous effects of interruptions in other domains (Walji et al., 2004). For instance, potential benefits in visual search tasks could be explored by implementing strategic interruptions, such as signaling alerts. These alerts could enhance efficiency and effectiveness in security-critical tasks such as baggage screening, where quick identification and processing are essential (Boskemper et al., 2022). Furthermore, interruptions could prove beneficial in human-assisting monitoring and management systems, such as those used in traffic control. For example, timely pop-ups could prompt operators to make necessary adjustments based on real-time data (Dahal et al., 2013). By taking this approach, a more nuanced range of hypotheses regarding the role of interruptions could be examined, moving beyond the commonly held view that they are primarily disruptive, toward a more comprehensive understanding that acknowledges their potential utility as well.

Our review identified a notable gap in the literature concerning the influence of individual differences on the effects of interruptions during search tasks. One study found that individuals with greater working memory capacity experienced less negative impact from interruptions on search accuracy, regardless of the duration of the interruption (Labonté and Vachon, 2021). This suggests that individual cognitive capacities might play a role in mitigating the adverse effects of interruptions. Moreover, while existing research suggests that individual expertise is associated with enhanced performance in visual search tasks (Robson et al., 2021; Wang et al., 2021), the question of whether expertise also contributes to more effective management of interruptions in visual search remains debated. For instance, when comparing the performance of experienced and novice radiologists in interpreting chest scans, Wynn et al. (2018) found no significant differences between the groups when their search was interrupted; both were equally affected, exhibiting extended scanning times and diminished response accuracy compared to the uninterrupted condition. These findings highlight the need for further research to clarify the potential moderating effects of experience and training on interruption management.

One limitation of our review is the deliberate focus on studies involving young and healthy adult populations. During our review process, we found and excluded one study that included children and one study with elderly participants. The first study, by Lleras et al. (2011), found that the ability to quickly resume searching after a brief interruption (i.e., rapid resumption) does not vary with age among children and adolescents aged 7 to 19 years. In the second study, Farrimond et al. (2006) observed that older adults, unlike younger adults, experienced a significant decline in cue detection following interruptions during a scene navigation task. They suggested that this decline could be related to a diminished capacity for self-initiated reinstatement of working memory in older age. Future research could benefit from incorporating a broader range of demographic and clinical populations to better understand how interruptions impact cognitive processes in these groups.

Our review underscores the complex nature of interruptions in visual search as we strive to establish an operational definition that captures the diversity noted in the literature. We consider that this definition was crucial for accurately categorizing the studies and developing a preliminary scheme intended as a starting point to deepen understanding of the phenomenon and facilitate comparisons across different research disciplines. Despite our efforts to define clear criteria, we are aware that our categorization scheme may be subject to critique and could require refinement or expansion in future research. For instance, parallels can be drawn between the paradigms discussed in this review and those of dual-tasks (Liesefeld et al., 2024) and task-switching (Kiesel et al., 2010). On the one hand, when search resumption is conditioned to the completion of an interruption that entails another task, this interruption can be regarded as a secondary task. On the other hand, while task-switching usually does not reflect visual search situations, it requires a quick alternation between different tasks and adaptation to the currently relevant task set. However, due to significant differences in execution and cognitive demands, studies employing these paradigms were deemed beyond the scope of our review.

In conclusion, we aimed to provide a nuanced perspective that emphasized the need for dedicated research and standardized methodologies, which are crucial for facilitating valid comparisons across studies and integrating the effects of interruptions into current visual search models. We also advocate for a reevaluation of the traditional view that interruptions are predominantly negative and encourage exploration of their potential benefits. This shift in perspective could significantly impact not only academic research but also offer broader real-world applications of these insights.

## Data availability statement

The original contributions presented in the study are included in the article/supplementary material, further inquiries can be directed to the corresponding author.

## Author contributions

AC-D: Conceptualization, Investigation, Methodology, Visualization, Writing – original draft, Writing – review & editing. SN: Investigation, Writing – review & editing, Methodology. CK: Writing – review & editing. IG: Writing – review & editing. MH: Conceptualization, Investigation, Resources, Supervision, Writing – review & editing, Funding acquisition.
